# Association Between Pre-Operative Fat Mass and Appendicular Lean Soft-Tissue Mass Before and One Year After Laparoscopic Sleeve Gastrectomy: A Sex-Stratified Body Composition Study

**DOI:** 10.3390/diagnostics16162656

**Published:** 2026-08-20

**Authors:** Yusuf Emre Altundal, Suleyman Buyukasik, Burak Kankaya, Mustafa Ozgul, Ege Ramadanoglu Turan, Halil Alis

**Affiliations:** 1 Department of General Surgery, Faculty of Medicine, Istanbul Aydin University, Istanbul 34295, Türkiye; suleymanbuyukask@gmail.com (S.B.); burakkankaya@gmail.com (B.K.); halilalis@gmail.com (H.A.); 2 A1 Diagnosis, Irvine, CA 92620, USA; ozgul@a1diagnosis.com; 3 Department of Nutrition and Dietetics, Istanbul Aydin University, Istanbul 34295, Türkiye; ege.ramadanoglu@hotmail.com

**Keywords:** bariatric surgery, bioimpedance analysis, appendicular lean soft tissue mass, fat mass, fat-free mass, sex differences

## Abstract

**Background/Objectives**: Total weight loss after laparoscopic sleeve gastrectomy (LSG) does not fully capture changes in body composition, particularly the loss of lean mass. We examined sex-specific changes in regional and whole-body composition before and one year after LSG. **Methods**: This retrospective single-center study included 121 adult patients (84 female, 37 male) who underwent LSG at Istanbul Aydin University Faculty of Medicine between May 2017 and February 2025. Multi-frequency segmental bioelectrical impedance analysis was performed pre-operatively and at one year post-operatively. Three ALM normalization indices (ALM/weight × 100, ALM/BMI, ALM/height^2^) were compared before and after surgery. Associations between pre-operative fat mass (FM) and ALM were examined using sex-stratified linear regression with and without adjustment for height, age, and sex via ordinary least squares residualization. **Results**: Pre- and post-operative values were compared. We found that BMI, fat-free mass (FFM), and FM decreased significantly in both sexes (all *p* < 0.001), with comparable total weight loss between females and males (*p* > 0.05). Males lost more absolute FFM and showed greater proportional lean mass loss in the limbs, while females showed greater proportional loss in the trunk. After adjusting for height, age, and sex, pre-operative FM was positively associated with ALM both pre-post-operatively (both *p* < 0.001). Patients with greater pre-operative FM lost more ALM over the surgical year. **Conclusions**: Body composition changes following laparoscopic sleeve gastrectomy in this cohort of 121 patients were metric-dependent. Patients with lower pre-operative adiposity may present with lower post-operative ALM one year after LSG.

## 1. Introduction

Over the past four decades, the global prevalence of obesity has risen markedly [1]. Bariatric surgical procedures have now become one of the foremost treatment options for severe obesity, providing both substantial weight loss and improvement of obesity-related cardiometabolic risk [2,3]. Among these procedures, laparoscopic sleeve gastrectomy (LSG) is currently the most frequently performed worldwide [4,5].

The obesity phenotype is not limited to an increase in fat mass (FM) alone; fat distribution and changes in muscle mass and body composition also play an important role in determining clinical outcomes [6]. Although age-related muscle loss had been clinically recognized as early as 1931 by Critchley [7], it was not until 1989 that Irwin H. Rosenberg formally named the condition sarcopenia at a meeting on the epidemiology of aging in Albuquerque, New Mexico [8]. He defined sarcopenia as the involuntary loss of skeletal muscle mass and consequently of strength [8]. In subsequent years, the concept was broadened to include not only loss of muscle mass but also loss of muscle strength and function [9].

Sarcopenic obesity was first defined by Robert N. Baumgartner as a clinical condition characterized by decreased muscle mass and/or muscle function accompanying increased FM [10]. Both sarcopenia and obesity have independently been linked to metabolic disorders, cardiovascular disease, and mortality, and sarcopenic obesity has been hypothesized to have a greater combined impact on these outcomes than either condition alone [11].

Bariatric surgery leads to substantial weight loss, which comprises losses of both FM and fat-free mass (FFM) [12]. In accordance with recent recommendations on body composition nomenclature [13], FFM is used throughout in preference to the historical term lean body mass, which has been shown to be chemically equivalent to FFM; lean soft tissue (LST) is used for compartments from which bone mineral has been excluded. Weight loss varies considerably between individuals, and total weight change alone does not fully reflect clinical outcomes. In the bariatric population, body composition assessment before and after surgery is therefore clinically important, particularly for identifying sarcopenia [2].

However, no clear consensus currently exists regarding the diagnosis of sarcopenia and sarcopenic obesity [9,14]. The methods used to measure muscle mass and the diagnostic threshold values applied differ substantially across studies, leading to diagnostic uncertainty in clinical practice [14]. Computed tomography (CT) and magnetic resonance imaging (MRI) are considered reference imaging techniques for body composition assessment but share several practical limitations. These include high cost and restricted availability outside research settings [15], as well as scanner bore and table weight capacities that may exclude patients with severe obesity, and reduced image quality due to beam attenuation, noise, and motion artifacts in this population [16]. CT involves ionizing radiation exposure [15,16]. Bioelectrical impedance analysis (BIA) is an indirect, equation-based method to calculate FM and FFM [17,18]. According to the recent ESPEN/EASO consensus, DXA is recommended as the first-choice method for body composition assessment, with BIA accepted as a valid second-choice alternative [15]. BIA is a widely used body composition method in routine clinical practice due to its portability, safety, and reproducibility [17], as well as its low cost and lack of radiation exposure compared with imaging-based techniques [18]. These features make it suitable for repeated assessment in outpatient settings.

Sarcopenic obesity is defined inconsistently across the literature, with substantial variation in the criteria, indices, and cutoff values applied [14], and this lack of consensus is particularly pronounced in the bariatric surgery population, where no standardized diagnostic approach currently exists [2]. The comparative performance of different indices, including appendicular lean soft tissue mass (ALM) normalized to body weight, BMI, and height^2^, as well as fat mass/fat-free mass (FM/FFM) ratio, has not been systematically evaluated pre- and post-operatively in a sex-stratified manner in this setting.

To address the above gap, we evaluated body composition in 121 patients using bioelectrical impedance analysis before and one year after LSG. Our study had three aims. First, we characterized sex-specific changes in regional and whole-body lean soft tissue mass and FM following surgery. Second, we evaluated ALM using three scaling approaches (body weight, BMI, and height^2^), with sex-stratified pre- and post-operative comparisons. Third, we examined whether pre-operative FM, after adjustment for height, age, and sex, was associated with ALM before surgery, one year after surgery, and the change in ALM over the first post-operative year, and whether these associations differed between male and female patients.

## 2. Materials and Methods

### 2.1. Study Design and Participants

This was a single-center, retrospective observational cohort study of adult patients who underwent laparoscopic sleeve gastrectomy (LSG) at Istanbul Aydin University Faculty of Medicine, Istanbul, Türkiye, between May 2017 and February 2025. A total of 121 patients with complete pre-operative and one-year post-operative bioelectrical impedance analysis (BIA) measurements were included in the analysis.

Inclusion criteria were a pre-operative BMI ≥ 40 kg/m^2^, a BMI ≥ 35 kg/m^2^ with comorbidities, or a BMI ≥ 35 kg/m^2^ with family history related to comorbidities. Complete pre-operative and one-year post-operative BIA measurements were required in all cases. Excluded were 42 patients with a BMI of 32–35 kg/m^2^ who underwent metabolic surgery for uncontrolled type 2 diabetes mellitus, 37 patients who underwent gastric bypass surgery, 23 patients who underwent revision surgery, 1 patient whose procedure was converted to open surgery, and 71 patients for whom one-year BIA data were missing. Of these exclusions, 103 patients were excluded by design because they did not meet the inclusion criteria or did not undergo laparoscopic sleeve gastrectomy. The remaining 71 patients met the inclusion criteria and underwent LSG but had no one-year post-operative BIA measurement within the study period. The baseline characteristics of the two groups are compared, overall and by sex, in Table S2. Overall, the two groups did not differ in sex distribution, pre-operative weight, BMI, FM, FFM or ALM (all *p* > 0.05); the patients without a one-year measurement were younger (*p* < 0.01) and taller (*p* < 0.05), and the height difference reflects the sex composition of the two groups and was not significant within either sex. Among women, age was the only characteristic that differed (*p* < 0.05). Among men, age did not differ, but those without a one-year measurement had lower pre-operative weight (*p* < 0.05), BMI (*p* < 0.01) and FM (*p* < 0.01). The resulting potential for selection bias is discussed in Section 4.10. The cohort was 69% female (84 women, 37 men). The patient flow diagram is shown in Figure S1.

The study protocol was approved by the Clinical Research Ethics Committee of Istanbul Aydin University Faculty of Medicine (Decision No. 47/2024, Date: 5 June 2024). Because of the retrospective design, the requirement for written informed consent was waived by the Ethics Committee. The study was conducted in accordance with the 1964 Declaration of Helsinki.

### 2.2. Surgical Procedure and Perioperative Care

All patients underwent comprehensive pre-operative evaluation, including anesthetic, endocrine, and psychiatric assessment, as well as routine esophagogastroduodenoscopy. The surgical technique used in our institution has been described in detail previously by our group [19]. All procedures were performed laparoscopically; one patient who required intraoperative conversion to open surgery was excluded from the analysis.

### 2.3. Body Composition Measurement

Body composition measurements were performed pre-operatively and at one year post-operatively using a multi-frequency segmental bioelectrical impedance analysis device (Tanita MC-980, Tanita Corporation, Tokyo, Japan). Measurements were obtained according to manufacturer protocols and performed in the morning, in a fasted state, after bladder emptying, and at rest without vigorous physical activity. Standardization was applied to minimize measurement variation related to hydration status.

The following parameters were obtained from the device output at each timepoint: body weight, body mass index, FM, FFM, body fat percentage, and regional segmental lean soft tissue mass for the trunk, both arms and both legs. Regional segmental FM was also recorded for each body compartment. Within the device’s model, FFM comprises lean soft tissue mass and an estimated bone mass; the manufacturer defines the segmental output as bone-free lean tissue mass. These segmental values therefore correspond to lean soft tissue (LST), comprising skeletal muscle together with non-muscle organs, skin, connective tissue and body fluids, rather than to skeletal muscle mass [13,15]. Following Heymsfield et al. [13], ALM is defined as the sum of the lean soft tissue mass of both arms and both legs. The abbreviation ALM is retained for consistency with the ESPEN/EASO consensus statement and the wider sarcopenia literature; it should not be equated with appendicular skeletal muscle mass, and cutpoints derived from dual-energy X-ray absorptiometry are therefore not directly transferable to these values. Age was recorded at the time of pre-operative measurement, and sex was recorded as a binary variable (male, female).

### 2.4. Statistical Analysis

Normally distributed continuous variables are presented as mean ± standard deviation (SD). Continuous variables that departed from normality are presented as median (interquartile range, IQR). The Shapiro–Wilk test was used to assess normality in each sex separately, and it determined both the summary statistic reported in Table 1 and the appropriate statistical test for each comparison; it was applied to pre-operative and post-operative values for each group and to difference scores (post-operative minus pre-operative) where appropriate.

Among the baseline variables in Table 1, age and height were normally distributed in both sexes (*p* > 0.05). Post-operative BMI and post-operative FM were non-normally distributed in both sexes (*p* < 0.05). Pre-operative weight, post-operative weight, total weight loss percentage, pre-operative BMI, pre-operative FM, pre-operative and post-operative FFM, and pre-operative and post-operative ALM were non-normally distributed in women (*p* < 0.05) but normally distributed in men (*p* > 0.05); these variables are reported as median (IQR) in all columns, which keeps the columns of a row directly comparable, and each row therefore uses a single summary statistic.

**Figure 1:**
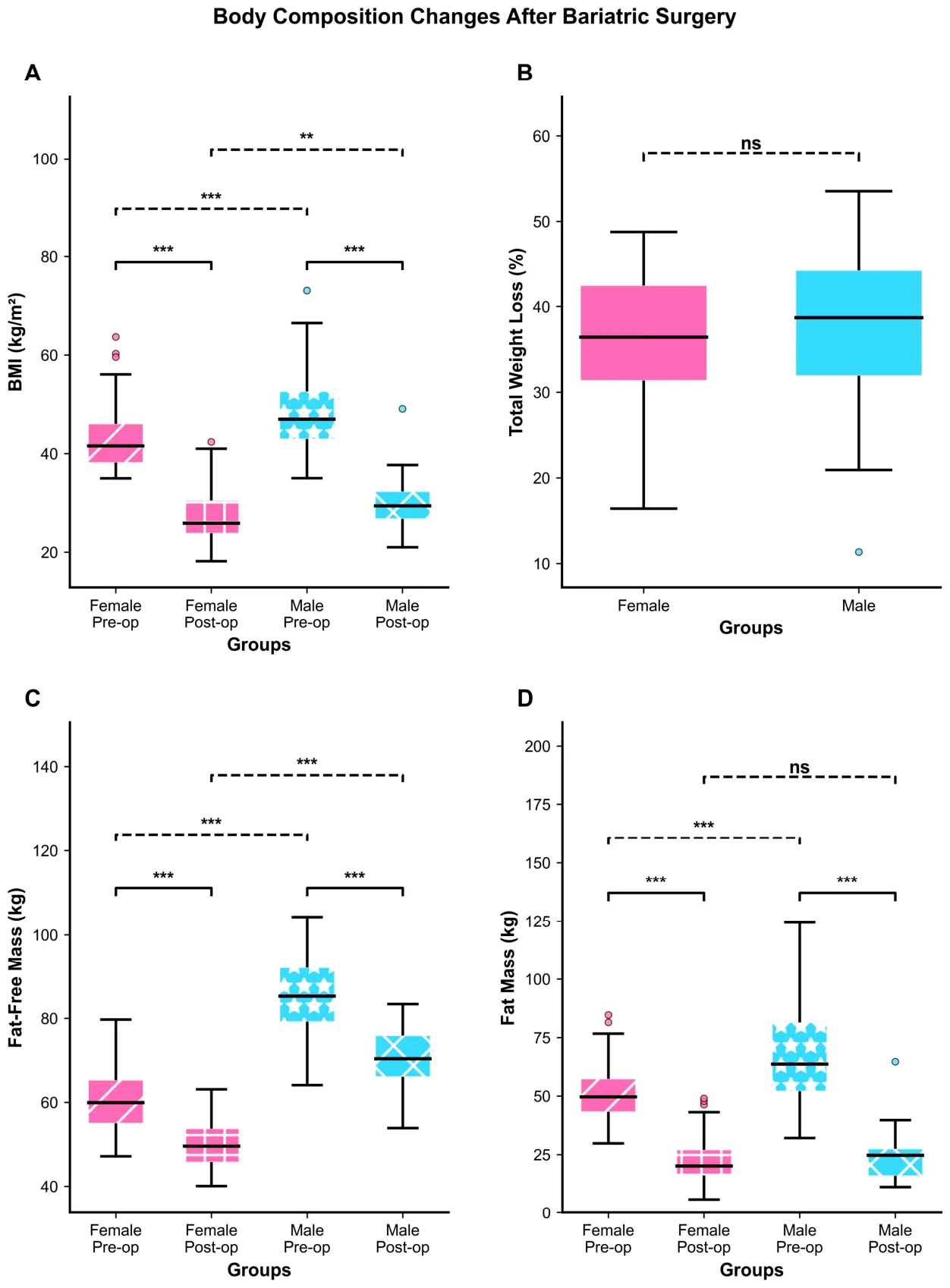
Body composition changes after LSG stratified by sex. (**A**) Body mass index. (**B**) Total weight loss percentage. (**C**) Fat-free mass. (**D**) Fat mass. Boxes span the interquartile range, the horizontal line marks the median, whiskers extend to 1.5 times the interquartile range and points beyond are plotted individually. Solid brackets indicate within-sex comparisons; dashed brackets indicate between-sex comparisons. ** *p* < 0.01; *** *p* < 0.001; ns: not significant.

**Figure 2:**
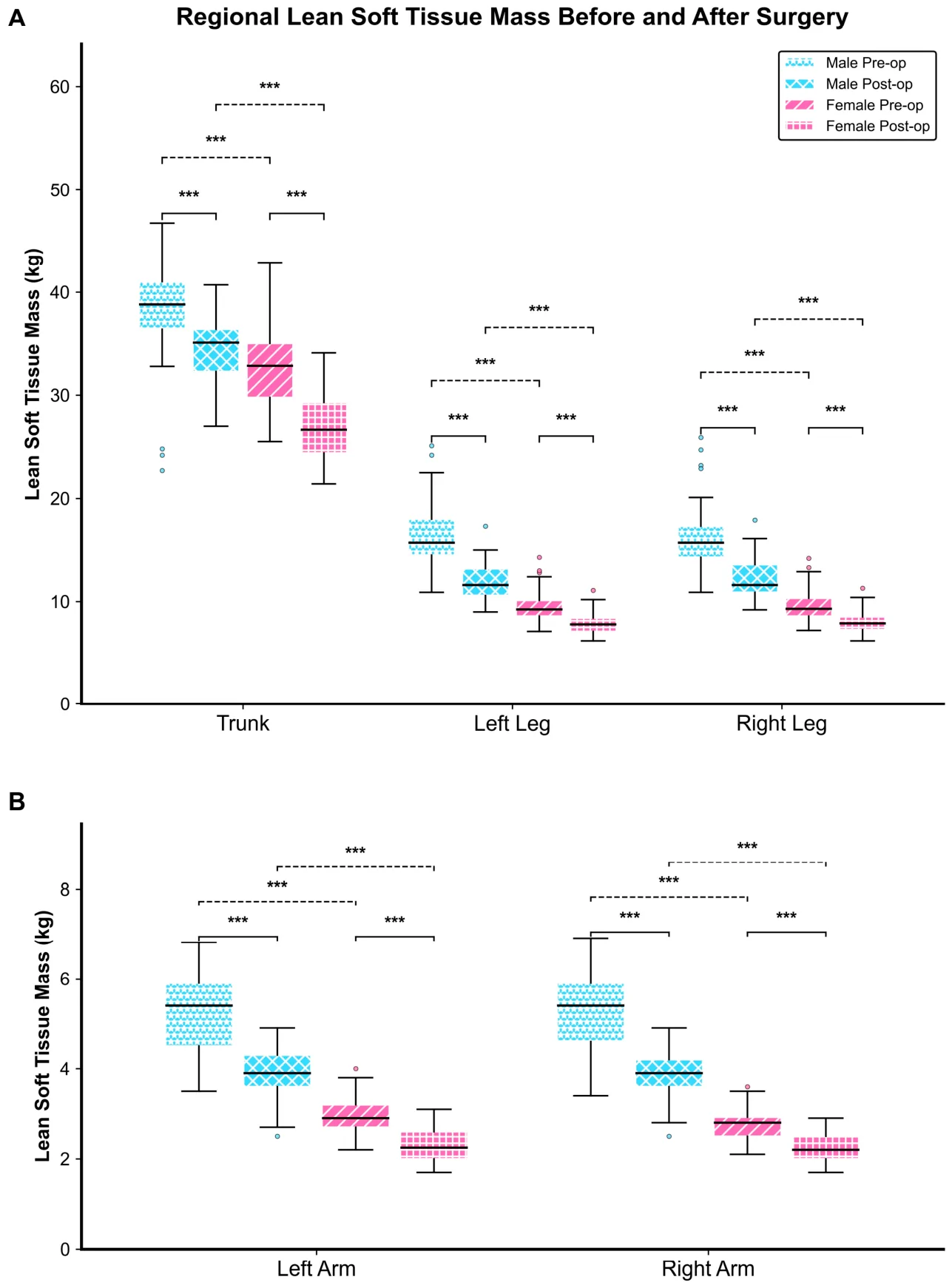
Regional lean soft tissue mass distribution (kg) before and after LSG by body region and sex. (**A**) Trunk and legs. (**B**) Arms, shown on a separate y-axis scale because their values are roughly an order of magnitude smaller. Boxes span the interquartile range, the horizontal line marks the median, whiskers extend to 1.5 times the interquartile range and points beyond are plotted individually. Solid brackets indicate within-sex comparisons; dashed brackets indicate between-sex comparisons. *** *p* < 0.001.

**Figure 3:**
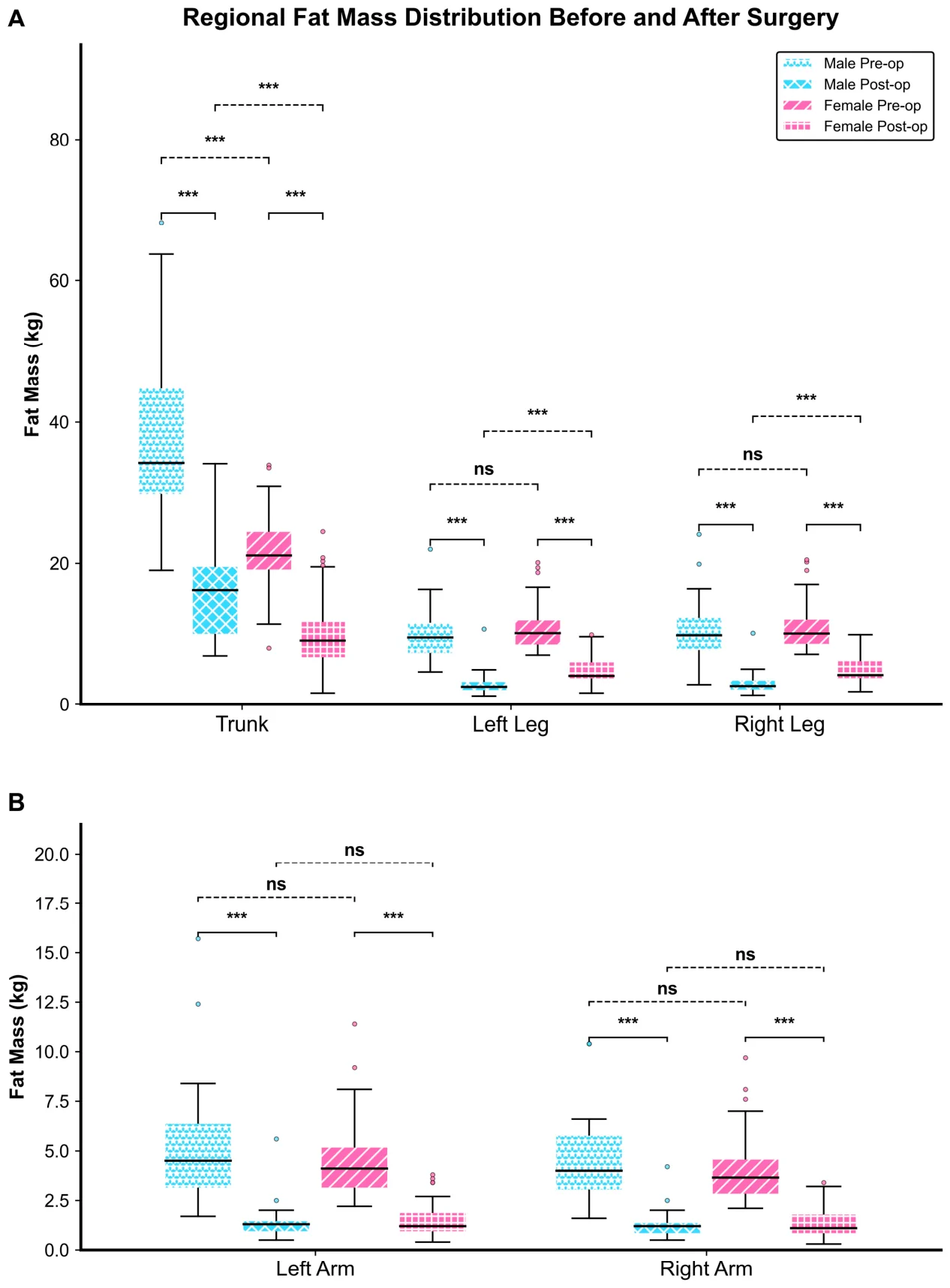
Regional fat mass distribution (kg) before and after LSG by body region and sex. (**A**) Trunk and legs. (**B**) Arms, shown on a separate y-axis scale because their values are roughly an order of magnitude smaller. Boxes span the interquartile range, the horizontal line marks the median, whiskers extend to 1.5 times the interquartile range and points beyond are plotted individually. Solid brackets indicate within-sex comparisons; dashed brackets indicate between-sex comparisons. *** *p* < 0.001; ns: not significant.

**Figure 4:**
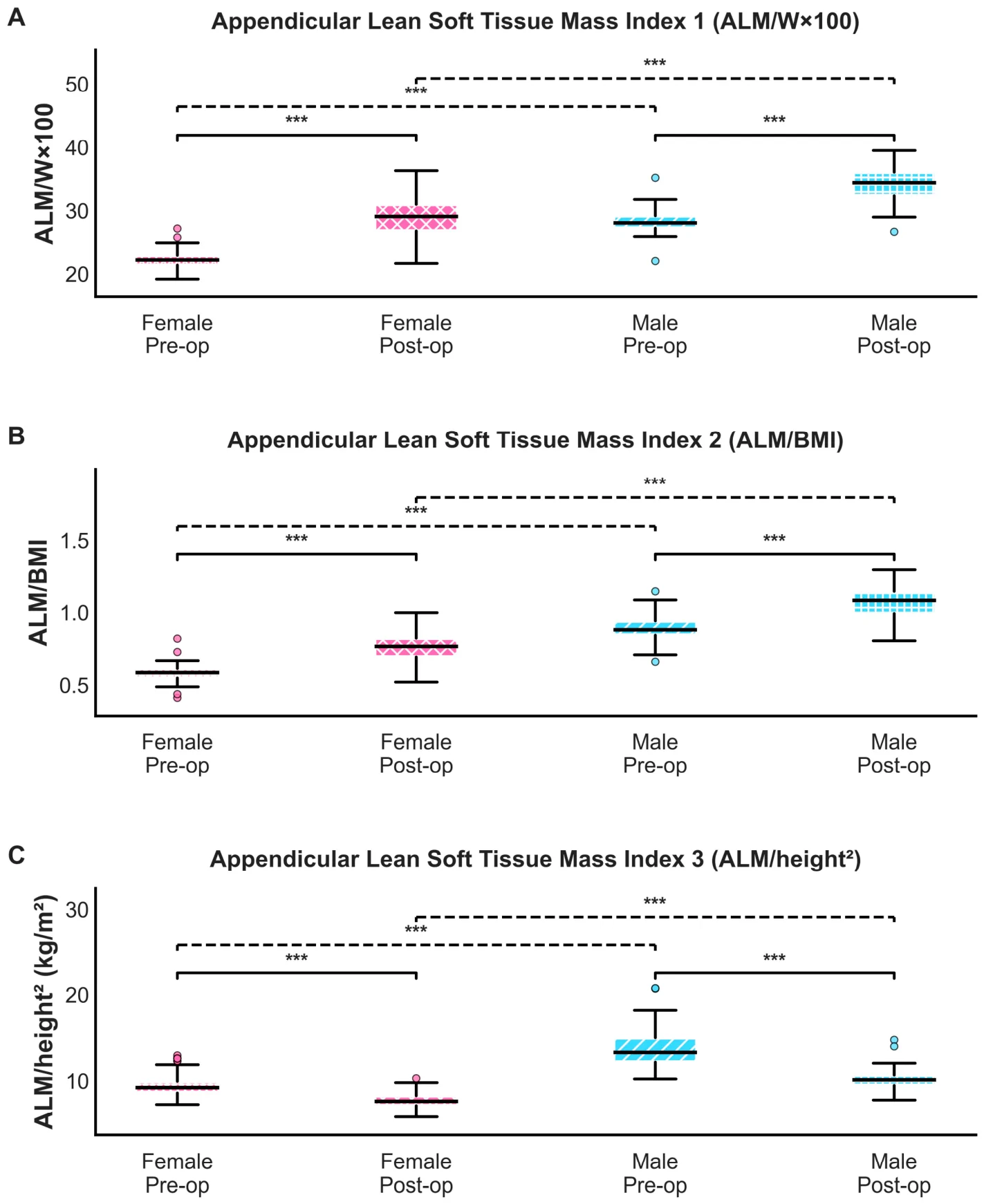
Changes in three ALM indices before and after LSG, stratified by sex. (**A**) ALM/W × 100. (**B**) ALM/BMI. (**C**) ALM/height^2^ (kg/m^2^). Boxes span the interquartile range, the horizontal line marks the median, whiskers extend to 1.5 times the interquartile range and points beyond are plotted individually. Solid brackets indicate within-sex comparisons; dashed brackets indicate between-sex comparisons. *** *p* < 0.001.

**Figure 5:**
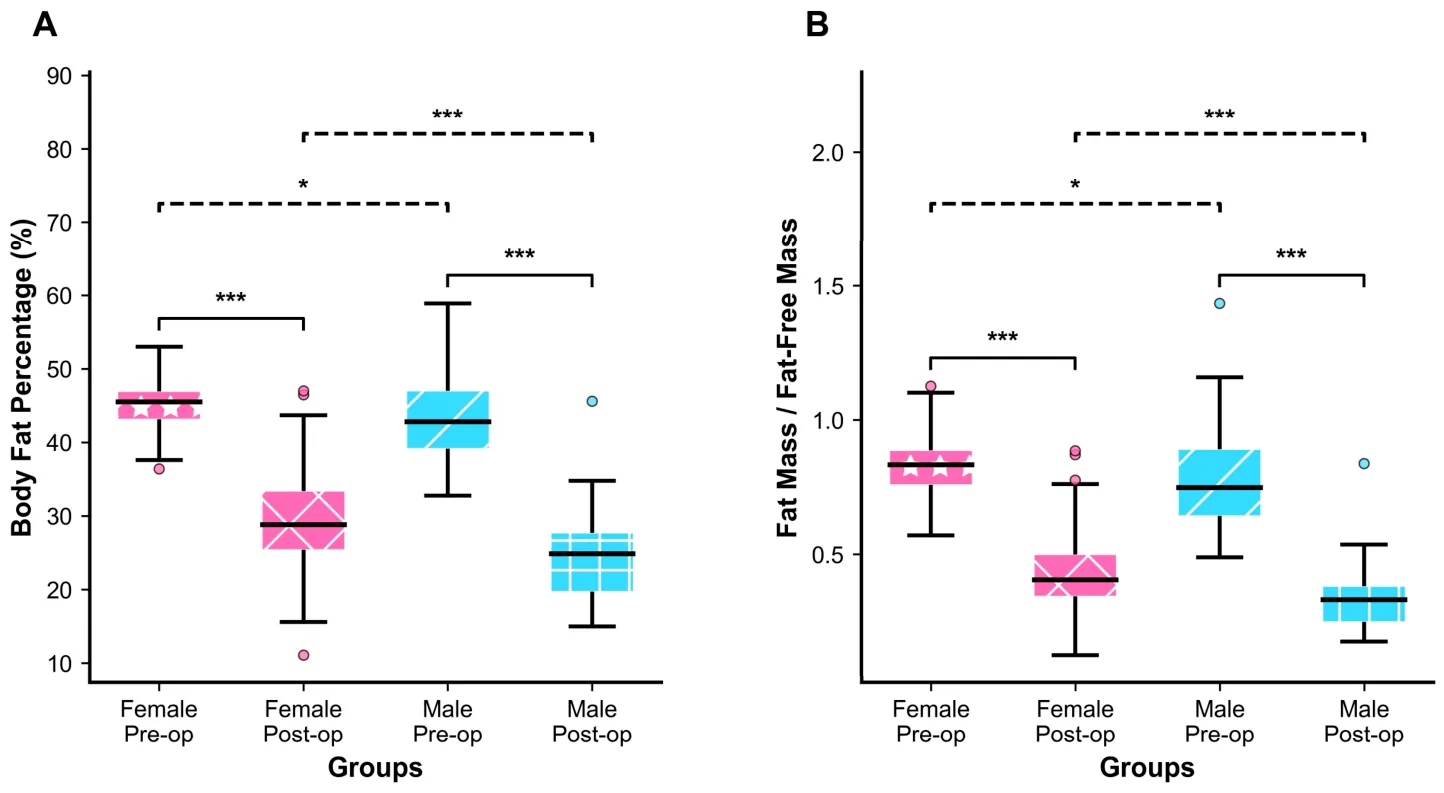
Body fat percentage (**A**) and FM/FFM ratio (**B**) before and after LSG stratified by sex. Boxes span the interquartile range, the horizontal line marks the median, whiskers extend to 1.5 times the interquartile range and points beyond are plotted individually. Solid brackets indicate within-sex comparisons; dashed brackets indicate between-sex comparisons. * *p* < 0.05; *** *p* < 0.001.

**Figure 6:**
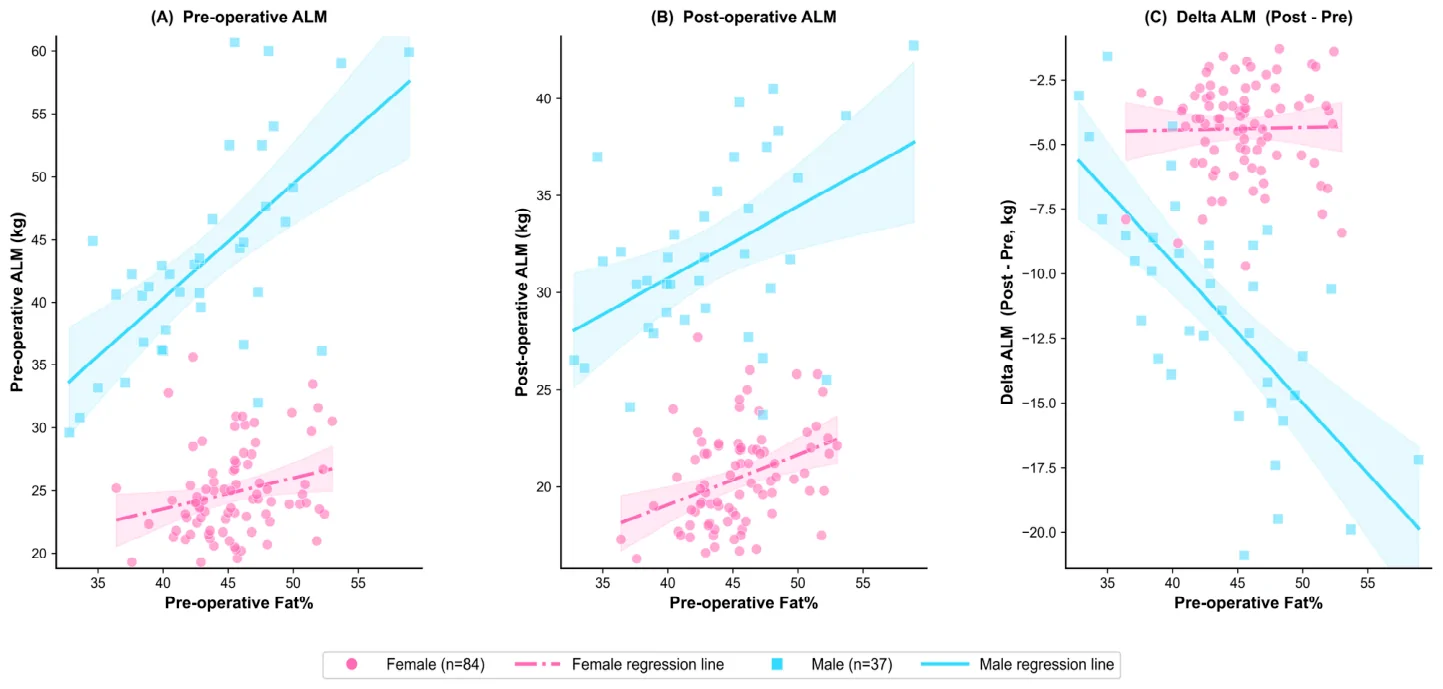
Unadjusted, sex-stratified relationship between pre-operative body fat percentage and ALM: (**A**) pre-operative, (**B**) post-operative, and (**C**) change in ALM. Lines represent linear regression fits with 95% confidence intervals.

**Figure 7:**
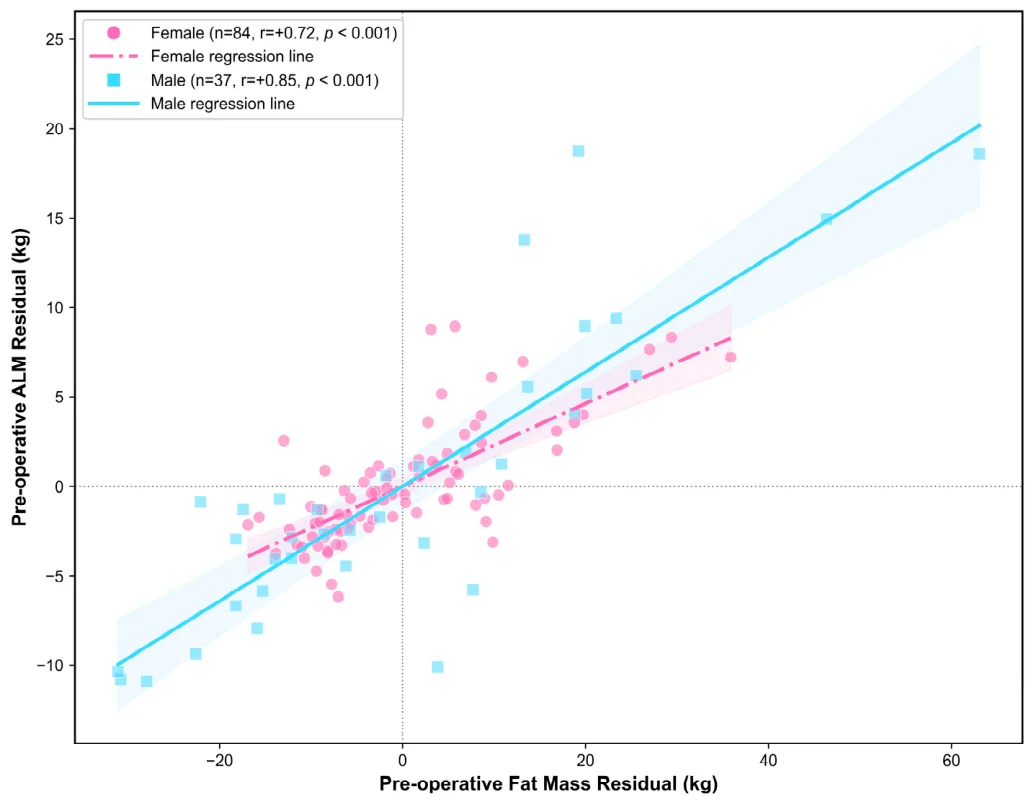
Sex-stratified relationship between pre-operative FM and pre-operative ALM, with both variables residualized on height, age, and sex. Axes show deviations from model estimates: zero denotes the value estimated for a patient of that height, age, and sex in this cohort; positive and negative values indicate more or less than estimated. Lines represent sex-specific linear regression fits with 95% confidence intervals.

**Figure 8:**
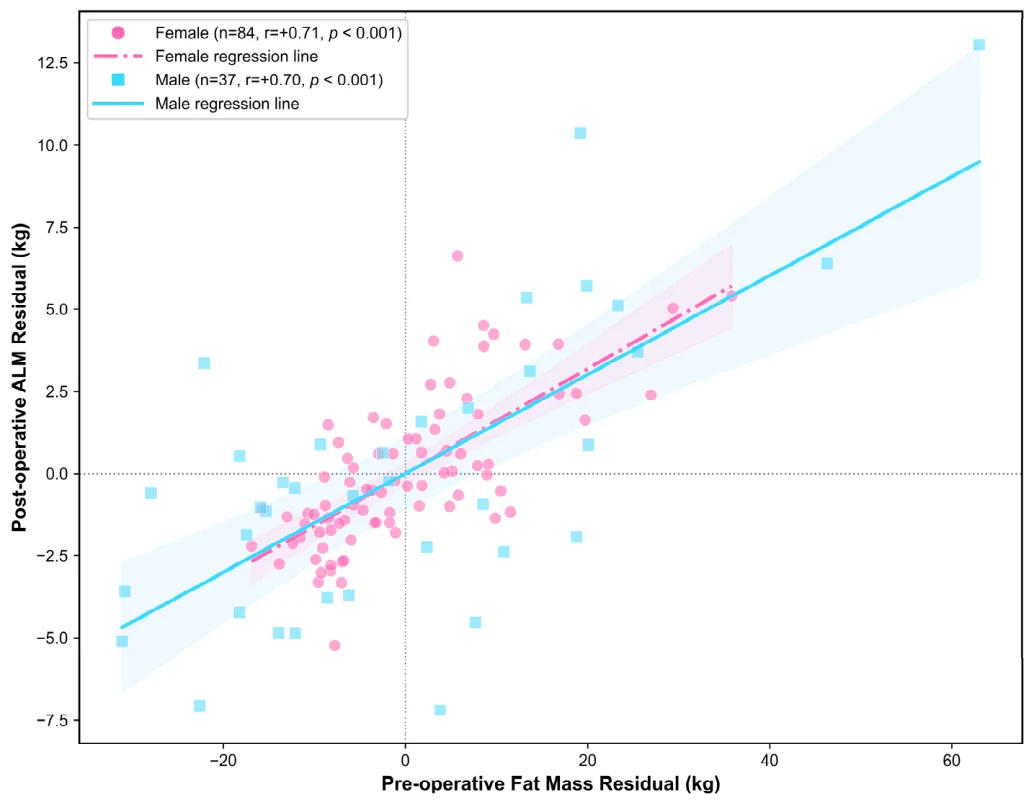
Sex-stratified relationship between pre-operative FM and post-operative ALM, with both variables residualized on height, age, and sex. Axes and interpretation as in Figure 7.

**Figure 9:**
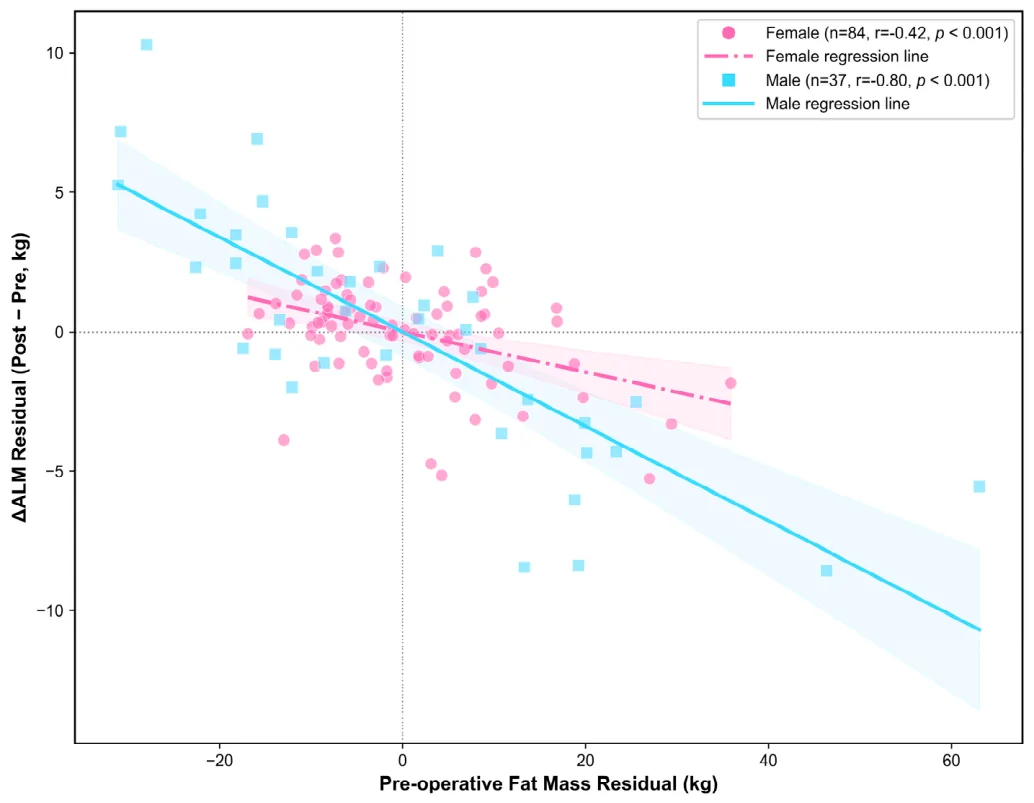
Sex-stratified relationship between pre-operative FM and change in ALM after surgery, with both variables residualized on height, age, and sex. Axes and interpretation as in Figure 7.

For pre-operative to post-operative comparisons within each sex, the paired *t*-test was used when the difference scores were normally distributed and the Wilcoxon signed-rank test was used otherwise. For between-sex comparisons of independent measurements, Welch’s independent *t*-test was used when both groups were normally distributed, and the Mann–Whitney U test was used otherwise. Figure 1, Figure 2, Figure 3, Figure 4 and Figure 5 are box plots, in which the box spans the interquartile range, the horizontal line marks the median, the whiskers extend to 1.5 times the interquartile range and points beyond that are plotted individually. In Figure 6, Figure 7, Figure 8 and Figure 9, shaded bands represent the 95% confidence interval of the sex-stratified regression line. The statistical test applied to each comparison reported in Table 1 and Figure 1, Figure 2, Figure 3, Figure 4 and Figure 5 is given in Table S1.

The association between pre-operative body fat and ALM was examined using two approaches.

First, unadjusted (simple bivariate) associations, without accounting for differences in height, age, or sex, were examined using Pearson correlation coefficients and sex-stratified linear regression (Figure 6); body fat percentage, pre-operative ALM (in kg), post-operative ALM (in kg), and change in ALM (in kg) were utilized in these analyses.

Second, adjusted analyses were performed in which pre-operative FM (in kilograms) and ALM were calculated to account for potential confounding effects of age, height, and sex, thereby isolating the fat–muscle relationship from these confounding variables (Figure 7, Figure 8 and Figure 9). For the adjusted analyses, both variables were residualized on height, age, and sex using ordinary least squares (OLS) regression. The residuals were calculated as the observed value minus the value estimated by the model: (1) pre-operative ALM minus estimated pre-operative ALM, (2) post-operative ALM minus estimated post-operative ALM, (3) change in ALM minus estimated change in ALM, and (4) pre-operative FM minus estimated pre-operative FM. Each residual is a patient’s deviation from the value estimated for a patient of the same height, age, and sex in this cohort. The relationship between adjusted FM and adjusted ALM was then assessed using Pearson correlation coefficients and sex-stratified linear regression. This adjusted framework was applied to three outcomes: pre-operative ALM (Figure 7), post-operative ALM (Figure 8), and the change in ALM over the surgical year (Figure 9).

In both the unadjusted and adjusted analyses, a statistical interaction term between sex and fat was included in the regression model to test whether the fat-ALM association differed between males and females. Because residual variance in ALM was greater in men than in women, interaction *p*-values were obtained using heteroscedasticity-consistent (HC3) robust standard errors, pre-specified as the primary inference for these tests.

All fat-ALM analyses (Figure 6, Figure 7, Figure 8 and Figure 9) were exploratory; no adjustment for multiple comparisons was applied, and *p*-values should be interpreted accordingly. A two-tailed *p*-value of less than 0.05 was considered statistically significant, with significance levels denoted as * *p* < 0.05, ** *p* < 0.01, and *** *p* < 0.001. All statistical analyses were performed using Python (version 3.13.6) with SciPy (version 1.16.3), statsmodels (version 0.14.6), pandas (version 2.3.3), NumPy (version 2.3.3) and matplotlib (version 3.10.8).

The study size was determined by the number of eligible patients with complete paired pre-operative and one-year measurements available during the study period. All analyses were stratified by sex, and the tests described above assume neither equal group sizes nor equal residual variances. A sensitivity power analysis indicated that, at α = 0.05 (two-tailed) and 80% power, the full cohort (*n* = 121) was able to detect a correlation of |r| ≥ 0.25 and a paired within-subject effect size of dz ≥ 0.26; the corresponding minimum detectable values were |r| ≥ 0.30 and dz ≥ 0.31 in women (*n* = 84) and |r| ≥ 0.45 and dz ≥ 0.47 in men (*n* = 37), with between-sex comparisons able to detect a standardized mean difference in d ≥ 0.56.

## 3. Results

### 3.1. Baseline Characteristics

A total of 121 patients (84 female, 37 male) with complete pre-operative and one-year post-operative data were included in the final analysis. Baseline demographic and anthropometric characteristics are summarized in Table 1.

Age did not differ significantly between the sexes. Male patients were significantly taller, and had significantly higher weight and BMI both before surgery and one year afterwards. Pre-operative FM and pre-operative ALM were also higher in male patients (both *p* < 0.001).

### 3.2. Body Composition Changes Following Laparoscopic Sleeve Gastrectomy

Figure 1 illustrates the changes in body composition parameters following LSG, stratified by sex (female, *n* = 84; male, *n* = 37). Patients with obesity demonstrated significant alterations across all measured parameters. Median values with interquartile ranges are given in Table 1.

In Figure 1A and Table 1, BMI in female patients decreased significantly post-operatively (*p* < 0.001). Male patients exhibited a similarly significant reduction in BMI (*p* < 0.001). BMI was higher in male than in female patients both before (*p* < 0.001) and one year after surgery (*p* < 0.01).

In Figure 1B and Table 1, total weight loss percentage was comparable between sexes, with no statistically significant difference between groups (*p* > 0.05).

In Figure 1C and Table 1, FFM (kg) decreased significantly in both groups after surgery (both *p* < 0.001). FFM (kg) was higher in male than in female patients at both timepoints (both *p* < 0.001).

In Figure 1D and Table 1, FM (kg) measurements revealed the most pronounced changes post-operatively. Female patients experienced a 59.5% decrease in FM (*p* < 0.001) and male patients a 61.2% decrease (*p* < 0.001). Pre-operative FM was higher in male patients (*p* < 0.001), whereas post-operative FM did not differ significantly between the sexes (*p* > 0.05).

Notably, while both sexes showed significant improvements, male patients exhibited larger absolute changes in BMI (median change −20.10 vs. −15.85 kg/m^2^, *p* < 0.01), FFM (−14.90 vs. −10.55 kg, *p* < 0.001) and FM (−44.60 vs. −29.65 kg, *p* < 0.001) compared to female patients, despite similar percentage total weight loss. The proportional loss of FFM did not differ significantly between the sexes (male 17.5% vs. female 17.3%, *p* > 0.05).

### 3.3. Regional Lean Soft Tissue Mass Distribution Changes

Figure 2 illustrates the regional lean soft tissue mass distribution analysis, which demonstrated significant changes across all body compartments following LSG in both male (*n* = 37) and female (*n* = 84) patients. Median values with interquartile ranges for each region and timepoint are given in Table 2.

In Figure 2A and Table 2, male patients exhibited a significant decrease in trunk lean soft tissue mass post-operatively (*p* < 0.001), a 9.54% reduction. Female patients showed a more pronounced reduction in trunk lean soft tissue mass (*p* < 0.001), an 18.87% reduction.

In Figure 2A and Table 2, lower extremity lean soft tissue mass decreased significantly in both sexes. In male patients, left leg lean soft tissue mass decreased by 26.11% and right leg lean soft tissue mass by 26.11% (both *p* < 0.001). Female patients demonstrated reductions of similar direction but smaller magnitude, with left leg lean soft tissue mass decreasing by 15.68% and right leg lean soft tissue mass by 15.05% (both *p* < 0.001).

In Figure 2B and Table 2, upper extremity measurements revealed similar patterns of lean soft tissue mass reduction. In male patients, left arm lean soft tissue mass decreased by 27.78% and right arm lean soft tissue mass by 27.78% (both *p* < 0.001). Female patients showed corresponding reductions in left arm lean soft tissue mass of 22.41% and right arm lean soft tissue mass of 21.43% (both *p* < 0.001).

Lean soft tissue mass was higher in male than in female patients in every region at both timepoints (all *p* < 0.001).

The percentage decrease in lean soft tissue mass varied between sexes across body regions, with male patients showing greater proportional losses in the limbs (left arm *p* < 0.05; other limbs *p* < 0.001) and female patients showing greater proportional loss in the trunk (*p* < 0.001). The greater trunk loss in female patients was also present in absolute terms (*p* < 0.001).

### 3.4. Regional FM Distribution Changes Following LSG

Figure 3 illustrates the regional FM distribution analysis, which demonstrated significant reductions across all body compartments following LSG in both male (*n* = 37) and female (*n* = 84) patients (*p* < 0.001 for all regions). Median values with interquartile ranges for each region and timepoint are given in Table 2.

In Figure 3A and Table 2, male patients exhibited the largest absolute trunk FM reduction (*p* < 0.001), a 52.63% reduction. Female patients showed a similarly significant reduction in trunk FM (*p* < 0.001), a 57.11% reduction.

In Figure 3A and Table 2, lower extremity FM decreased substantially in both sexes. In male patients, left leg FM decreased by 73.68% and right leg FM by 73.47% (both *p* < 0.001). Female patients demonstrated corresponding reductions, with left leg FM decreasing by 59.90% and right leg FM by 58.71% (both *p* < 0.001).

In Figure 3B and Table 2, upper extremity FM also showed marked reductions following surgery. In male patients, left arm FM decreased by 71.11% and right arm FM by 70.00% (both *p* < 0.001). Female patients showed corresponding reductions in left arm FM of 70.73% and right arm FM of 69.86% (both *p* < 0.001).

Between the sexes, trunk FM was higher in male patients both before and after surgery (both *p* < 0.001). Leg FM did not differ before surgery (*p* > 0.05) but was lower in male patients at one year (both legs *p* < 0.001). Arm FM did not differ between the sexes at either timepoint (all *p* > 0.05).

Male patients exhibited greater proportional FM reductions in the lower extremities than female patients (both legs *p* < 0.001), whereas proportional reductions in the trunk and upper extremities did not differ significantly between the sexes (all *p* > 0.05). The absolute trunk FM reduction was nevertheless greater in male patients (*p* < 0.001). Overall, the largest relative reductions in FM were observed in the limbs rather than the trunk, exceeding 69% in the upper extremities of both sexes and in the male lower extremities.

### 3.5. Assessment of Changes in Appendicular Lean Soft Tissue Mass Indices Following LSG

In Figure 4, three different appendicular lean soft tissue mass indices were evaluated to assess changes in muscle mass following LSG in both male (*n* = 37) and female (*n* = 84) patients. Median values with interquartile ranges are given in Table 2.

#### 3.5.1. Appendicular Lean Soft Tissue Mass Index 1 (ALM/W × 100)

Appendicular lean soft tissue mass as a percentage of total body weight (ALM/W × 100) showed significant differences between pre- and post-operative measurements in both sexes. In male patients, ALM/W × 100 increased significantly post-operatively (*p* < 0.001). Female patients demonstrated a similar significant increase (*p* < 0.001). Significant differences were also observed between male and female patients both pre-operatively (*p* < 0.001) and post-operatively (*p* < 0.001).

#### 3.5.2. Appendicular Lean Soft Tissue Mass Index 2 (ALM/BMI)

When appendicular lean soft tissue mass was normalized to BMI (ALM/BMI), both sexes showed significant increases following surgery. Male patients’ ALM/BMI increased significantly post-operatively (*p* < 0.001). Female patients showed a similar pattern (*p* < 0.001). Sex-based differences were significant both before (*p* < 0.001) and after surgery (*p* < 0.001).

#### 3.5.3. Appendicular Lean Soft Tissue Mass Index 3 (ALM/height^2^)

In contrast to the other indices, ALM/height^2^ decreased significantly following surgery in both sexes. Male patients’ ALM/height^2^ decreased significantly post-operatively (*p* < 0.001). Female patients exhibited a similar decrease (*p* < 0.001). Significant differences between males and females persisted both pre-operatively (*p* < 0.001) and post-operatively (*p* < 0.001).

These findings collectively demonstrate that while appendicular lean soft tissue mass relative to height (ALM/height^2^) decreased following surgery, the relative proportion of lean soft tissue mass to weight (ALM/W × 100) and to BMI (ALM/BMI) increased significantly, reflecting a preferential reduction in FM compared to lean soft tissue mass during surgical weight loss.

### 3.6. Assessment of Body Fat Percentage and FM/FFM Ratio Following LSG

In Figure 5, body fat percentage and FM/FFM ratio were analyzed in both male (*n* = 37) and female (*n* = 84) patients following LSG. Median values with interquartile ranges are given in Table 2.

#### 3.6.1. Body Fat Percentage

In Figure 5A, body fat percentage showed significant reductions in both sexes following LSG. Female patients exhibited a decrease in body fat percentage (*p* < 0.001), representing a 36.6% relative reduction. Similarly, male patients demonstrated a more pronounced decrease (*p* < 0.001), representing a 41.8% relative reduction. These findings indicate substantial improvements in overall body composition. Body fat percentage was higher in female than in male patients before surgery (*p* < 0.05) and one year afterwards (*p* < 0.001), and the relative reduction was greater in male patients (*p* < 0.01).

#### 3.6.2. FM/FFM Ratio

In Figure 5B, the fat mass to fat-free mass ratio (FM/FFM) showed significant reductions in both sexes. In female patients, the FM/FFM ratio decreased significantly (*p* < 0.001), representing a 51.4% reduction. Male patients showed a similar pattern, with the FM/FFM ratio decreasing significantly (*p* < 0.001), representing a 55.7% reduction. The FM/FFM ratio was higher in female than in male patients before surgery (*p* < 0.05) and one year afterwards (*p* < 0.001), and the relative reduction was greater in male patients (*p* < 0.01).

### 3.7. Association of Pre-Operative Body Fat Percentage with Appendicular Lean Soft Tissue Mass Before Surgery, After Surgery, and the Change

The central question addressed by Figure 6 was: does the amount of fat a patient carries before surgery relate to their ALM pre-operatively, one year later, and the degree of ALM loss in between? Figure 6 presents three panels sharing the same x-axis (pre-operative fat percentage), allowing a direct, sex-stratified comparison of how pre-operative adiposity relates to ALM at each stage. The relationships shown are unadjusted and descriptive. Regression coefficients, correlation coefficients and 95% confidence intervals for all models in Figure 6, Figure 7, Figure 8 and Figure 9 are reported in Table 3.

#### 3.7.1. Pre-Operative ALM vs. Pre-Operative Fat%

Higher pre-operative fat percentage was associated with higher pre-operative ALM in both sexes (female *p* < 0.05; male *p* < 0.001). The sex-specific slopes differed significantly (interaction *p* < 0.01): each 1% increase in pre-operative body fat corresponded to roughly four times as much ALM in males as in females (+0.92 vs. +0.25 kg).

#### 3.7.2. Post-Operative ALM vs. Pre-Operative Fat%

One year after surgery, pre-operative fat percentage remained positively associated with ALM in both sexes (female *p* < 0.001; male *p* < 0.01). Unlike the pre-operative pattern, the slopes no longer differed between sexes (interaction *p* > 0.05): the association was similar in men and women (+0.37 vs. +0.26 kg per 1% fat).

#### 3.7.3. Delta ALM vs. Pre-Operative Fat%

Among females, no detectable association was found between pre-operative fat percentage and ALM loss at one year (*p* > 0.05). Among males, higher pre-operative fat percentage was strongly associated with greater ALM loss (−0.55 kg of ALM per 1% fat, *p* < 0.001). The sex interaction was highly significant (*p* < 0.001): in this fat-percentage analysis, the relationship between adiposity and ALM loss was detected only in men.

### 3.8. Association of Pre-Operative FM and Pre-Operative ALM After Removing the Effects of Height, Age, and Sex

The central question addressed by Figure 7 was whether pre-operative FM is associated with pre-operative ALM after adjusting for height, age, and sex. To remove these influences, pre-operative FM and pre-operative ALM were each regressed on height, age, and sex, and the residuals were calculated as the observed value minus the value estimated by the model: (1) pre-operative ALM minus estimated pre-operative ALM, and (2) pre-operative FM minus estimated pre-operative FM. Each residual is a patient’s deviation from the value estimated for a patient of the same height, age, and sex in this cohort; zero on each axis therefore marks that estimated value, positive residuals indicate more fat (or more ALM) than estimated, and negative residuals less. Model estimates are reported in Table 3.

The answer was yes. Patients carrying more fat than estimated also carried more ALM than estimated in both sexes (both *p* < 0.001), corresponding to approximately 0.29 kg of additional ALM per additional kilogram of fat overall. The sex-by-fat interaction (*p* < 0.01) indicates this relationship was significantly stronger in males than in females: for each additional kilogram of fat above the estimate, males carried approximately 0.32 kg of additional ALM versus 0.23 kg in females.

### 3.9. Association of Pre-Operative FM and Post-Operative ALM After Removing the Effects of Height, Age, and Sex

The central question addressed by Figure 8 was whether pre-operative FM is associated with post-operative ALM one year after surgery, after adjusting for height, age, and sex. To remove these influences, post-operative ALM and pre-operative FM were each regressed on height, age, and sex, and the residuals were calculated as the observed value minus the value estimated by the model: (1) post-operative ALM minus estimated post-operative ALM, and (2) pre-operative FM minus estimated pre-operative FM. Each residual is a patient’s deviation from the value estimated for a patient of the same height, age, and sex in this cohort; zero on each axis therefore marks that estimated value, positive residuals indicate more fat (or more post-operative ALM) than estimated, and negative residuals less.

The answer was yes, but attenuated. Patients carrying more fat than estimated still carried more ALM than estimated one year after surgery (both *p* < 0.001), corresponding to approximately 0.15 kg of additional post-operative ALM per additional kilogram of fat overall, about half the pre-operative association (approximately 0.29 kg in Figure 7). Unlike the pre-operative pattern, the sex-by-fat interaction was not significant (*p* > 0.05), indicating that this relationship did not differ between males and females, with similar slopes in females (0.16 kg) and males (0.15 kg).

### 3.10. Association of Pre-Operative FM and Change in ALM After Removing the Effects of Height, Age, and Sex

The central question addressed by Figure 9 was whether pre-operative FM is associated with the change in ALM after surgery (post-operative minus pre-operative), after adjusting for height, age, and sex. To remove these influences, the change in ALM and pre-operative FM were each regressed on height, age, and sex, and the residuals were calculated as the observed value minus the value estimated by the model: (1) change in ALM minus estimated change in ALM, and (2) pre-operative FM minus estimated pre-operative FM. Each residual is a patient’s deviation from the value estimated for a patient of the same height, age, and sex in this cohort; zero on each axis marks that estimated value. On the vertical axis, negative residuals indicate a greater ALM loss than estimated and positive residuals a smaller loss; on the horizontal axis, positive residuals indicate more pre-operative fat than estimated.

The answer was yes. Patients carrying more fat than estimated lost more ALM than estimated (both *p* < 0.001), corresponding to approximately 0.14 kg of additional ALM loss per additional kilogram of fat overall. The sex-by-fat interaction (*p* < 0.05) indicates this relationship was significantly stronger in males than in females: for each additional kilogram of fat above the estimate, males lost more than twice as much ALM as females (approximately 0.17 kg versus 0.07 kg).

## 4. Discussion

This study examined sex-specific changes in body composition following laparoscopic sleeve gastrectomy using segmental bioelectrical impedance analysis in 121 patients (84 females, 37 males). Three principal findings emerged. First, while total weight loss was comparable between sexes, the composition and regional distribution of that loss differed: males lost more absolute FFM and showed greater proportional lean mass loss in the limbs, whereas females showed greater proportional lean mass loss in the trunk. Second, commonly used body composition indices produced contradictory conclusions about post-operative muscle status depending on the choice of denominator: ALM/weight and ALM/BMI increased after surgery while ALM/height^2^ decreased, and the FM/FFM ratio improved because FM declined proportionally faster than lean mass, not through preservation of lean tissue. Third, pre-operative FM was positively associated with ALM both before surgery and one year afterward, yet was also associated with greater loss of ALM over the surgical year. Patients carrying more excess fat pre-operatively had more lean mass both before and one year after surgery, but lost more lean mass during that period. These findings highlight that the interpretation of body composition after laparoscopic sleeve gastrectomy is highly dependent on the metric used, the body region examined, and whether the analysis is stratified by sex.

### 4.1. Changes in Body Composition Parameters

Nuijten et al. [12] distinguish the lean compartments explicitly, defining FFM as containing bone tissue, water, organ tissue and skeletal muscle, and lean body mass as total body mass minus FM and bone mineral content; their lean body mass therefore corresponds to what is here termed lean soft tissue.

Nuijten et al. [12] reported pooled losses at 12 months after bariatric surgery of 8.13 kg for lean body mass and 8.23 kg for FFM in a meta-analysis of 59 studies. Alba et al. [20] found that FM decreased by 49% and total lean mass by 14% at 12 months after gastric bypass, with men losing more lean mass than women (−12 vs. −8 kg, *p* = 0.01). Nguyen et al. [21] similarly reported that FM loss was greater in males than females (−27.0 vs. −20.9 kg, *p* = 0.01) at 12 months after sleeve gastrectomy. In the present study, BMI, FFM, and FM all decreased significantly in both sexes (Figure 1, *p* < 0.001 for all). Total weight loss percentage was comparable between females (36.4%) and males (38.7%, *p* > 0.05), yet males exhibited larger absolute reductions in both FFM (−14.90 vs. −10.55 kg, *p* < 0.001) and FM (−44.60 vs. −29.65 kg, *p* < 0.001). The greater absolute FFM loss in males is consistent with the findings of Alba et al. [20]. Notably, our data show that proportional FFM loss was nearly identical between sexes (17.3% in females vs. 17.5% in males, *p* > 0.05), indicating that the greater absolute FFM loss in males is related to their higher pre-operative FFM. This divergence between absolute and proportional readings of the same data illustrates the metric-dependence of body composition.

### 4.2. Regional Lean Mass Distribution Changes

Alba et al. [20] reported that men lost more total lean mass than women after gastric bypass (−12 vs. −8 kg at 12 months, *p* = 0.01), and that ALM declined by 16% overall. Nuijten et al. [22] found in a cohort of 3596 patients that being male was a determinant of a greater proportion of weight loss as fat-free mass (%FFML/WL; β = 3.99, *p* < 0.001), and this remained significant after multivariate adjustment. Maïmoun et al. [23] reported lean tissue mass changes by region (upper limbs, trunk, lower limbs) at 12 months after sleeve gastrectomy, with the loss approximately 20% and homogeneous across all sites, but did not stratify by sex. In the present study, lean mass decreased significantly across all body regions in both sexes (Figure 2, *p* < 0.001 for all). Lean mass loss in our cohort was sex-dependent: males showed greater proportional losses in the limbs (~26–28% in arms and legs), while females showed greater proportional loss in the trunk (18.9% vs. 9.5% in males). The proportional finding by Nuijten et al. [22] is consistent with the greater proportional limb loss we observed in males, while the larger absolute loss reported by Alba et al. [20] partly reflects men’s higher baseline lean mass. Neither study reported region-specific lean mass changes by sex. Our data extend these findings by providing sex-stratified, region-specific lean mass changes.

### 4.3. Regional FM Distribution Changes

Tałałaj et al. [24] assessed fat distribution in 155 patients (117 women, 38 men) after laparoscopic sleeve gastrectomy and found that subcutaneous fat decreased by 46% while abdominal fat decreased by 38% at 12 months. Total FM loss was similar between males and females (*p* = 0.81), but males lost significantly more abdominal fat (−6.7 vs. −4.6 kg, *p* = 0.01), while subcutaneous fat loss was comparable (−18.3 kg in males vs. −19.1 kg in females, *p* = 0.70). In the present study, FM decreased significantly across all body regions in both sexes (Figure 3, *p* < 0.001 for all). Males exhibited greater proportional fat reductions in the lower extremities compared to females, whereas trunk and upper extremity reductions were comparable between the sexes (trunk 52.6% in males vs. 57.1% in females). The largest relative reductions were observed in the limbs rather than the trunk, exceeding 69% in the upper extremities of both sexes and in the male lower extremities. While both our findings and those of Tałałaj et al. [24] confirm that regional fat loss after sleeve gastrectomy is sex-dependent, the compartmental pattern differs: Tałałaj et al. [24] found the male advantage in central (abdominal) fat, whereas in our cohort it was in the lower extremities, the only region where the male advantage reached significance (arms: 70–71% in males vs. 70–71% in females; legs: 73–74% in males vs. 59–60% in females). This discrepancy may reflect differences related to cohort characteristics such as age, genetics, ethnicity and diet types; further investigation is therefore warranted.

### 4.4. Changes in ALM Indices

Ozeki et al. [25] reported that relative skeletal muscle mass (MM/BW) increased from 0.29 to 0.36 (*p* < 0.001) at 12 months after laparoscopic sleeve gastrectomy, despite a decrease in absolute skeletal muscle mass from 33.4 to 29.0 kg (*p* = 0.03). Alba et al. [20] reported that ALM declined by 16% after gastric bypass, yet relative muscle strength (strength/BMI) increased by 32%, demonstrating the same denominator-driven divergence. Kim et al. [26] demonstrated that height-adjusted, weight-adjusted, and BMI-adjusted muscle mass indices yield different prevalence estimates and clinical classifications for sarcopenia, highlighting that the choice of denominator fundamentally alters interpretation. In the present study, ALM/weight and ALM/BMI both increased significantly after surgery in both sexes (Figure 4A,B), while ALM/height^2^ decreased significantly (Figure 4C). This divergent pattern is consistent with the findings of Ozeki et al. [25] and Alba et al. [20], and is explained by the denominator: weight and BMI decreased faster than ALM, causing weight- and BMI-normalized indices to rise, whereas height remained constant, allowing ALM/height^2^ to reflect the true direction of absolute muscle loss. These results underscore the warning of Kim et al. [26] that the choice of normalization index can produce opposite conclusions about muscle status after bariatric surgery.

### 4.5. Body Fat Percentage and FM/FFM Ratio

Ozeki et al. [25] reported that body fat percentage decreased from 48.1% to 33.4% at 12 months after laparoscopic sleeve gastrectomy in Japanese patients, while relative skeletal muscle mass (MM/BW) increased from 0.29 to 0.36 (*p* < 0.001), despite a decrease in absolute muscle mass. Nakaguchi et al. [27] similarly found that the skeletal muscle mass to fat mass ratio (SMM/FM) peaked at 12 months post-LSG before declining at 24 months. In the present study, body fat percentage decreased significantly in both sexes, with males achieving a greater relative reduction than females (Figure 5A). The FM/FFM ratio decreased substantially in both sexes (Figure 5B), consistent with the improvements in relative body composition reported by Ozeki et al. [25] and Nakaguchi et al. [27]. However, these ratio-based improvements should be interpreted with caution: they occur primarily because FM decreases faster than lean mass, not because muscle mass increased. The absolute loss of lean mass documented in Figure 1 and Figure 2 confirms that the improvement in FM/FFM ratio reflects a differential rate of loss rather than a genuine gain in muscle health.

### 4.6. Pre-Operative Fat–ALM Relationship

Janssen et al. [28] documented that men carry more skeletal muscle than women, with the greatest sex differences in the upper body, and that the contribution of skeletal muscle to weight gain decreases at higher body weights. The ESPEN/EASO consensus [15] recognized that excess adiposity and low muscle mass can coexist in sarcopenic obesity but did not address whether FM positively associated with muscle mass in pre-surgical populations. In the present study, patients with greater pre-operative fat carried greater ALM pre-operatively. In the unadjusted analysis (Figure 6A), this association was present in both sexes (female *p* < 0.05; male *p* < 0.001), with males showing a steeper slope (interaction *p* < 0.01). After adjusting for height, age, and sex (Figure 7), the relationship strengthened, with males again showing a stronger association than females (*p* < 0.01). These findings are consistent with the sex differences reported by Janssen et al. [28] and extend the ESPEN/EASO framework by demonstrating that in patients with severe obesity, higher adiposity is associated with higher ALM, and that this association differs by sex.

### 4.7. Post-Operative Attenuation and Disappearance of Sex Interaction

Nuijten et al. [12] reported pooled lean body mass loss at 12 months post-surgery in a meta-analysis, with 55% of this loss occurring within the first 3 months. Pekař et al. [29] similarly documented significant reductions in ALM index at 24 months after bariatric surgery. However, neither study examined whether the magnitude of post-operative ALM loss is related to pre-operative FM, or whether the fat-ALM relationship differs by sex over time. In the present study, one year after surgery, the positive association between pre-operative fat and post-operative ALM persisted but was attenuated in both the unadjusted (Figure 6B) and adjusted (Figure 8) analyses. The sex difference in slope that was significant pre-operatively (Figure 7, interaction *p* < 0.01) disappeared post-operatively (Figure 8, interaction *p* > 0.05). The unadjusted analysis showed the same pattern (Figure 6A interaction *p* < 0.01; Figure 6B interaction *p* > 0.05). Our findings extend these reports by showing that post-operative ALM loss is related to pre-operative FM, and that the sex-differential pattern present pre-operatively (Figure 6A and Figure 7) is no longer detectable one year after surgery (Figure 6B and Figure 8).

### 4.8. Sex-Differential ALM Loss

Nguyen et al. [21] examined sex differences in body composition changes at 12 months after laparoscopic sleeve gastrectomy and found that males lost significantly more skeletal muscle mass than females (−6.2 vs. −3.9 kg, *p* = 0.01). Janssen et al. [28] established that men carry more skeletal muscle than women (*p* < 0.001), with greater sex differences in the upper body (*p* < 0.01). In the present study, the change analysis revealed why the sex interaction disappeared post-operatively. In the adjusted analysis (Figure 9), males lost significantly more ALM per kilogram of excess pre-operative fat than females (interaction *p* < 0.05). In the unadjusted analysis (Figure 6C), no association between pre-operative fat percentage and ALM loss was detected in females (*p* > 0.05), while males showed a strong negative association (*p* < 0.001; interaction *p* < 0.001). The greater absolute muscle loss in males is consistent with the sex differences reported by Nguyen et al. [21] and with men having more adiposity-associated muscle available to lose as documented by Janssen et al. [28].

### 4.9. Metric Choice: Fat Percentage vs. FM in kg

Kim et al. [26] demonstrated that height-adjusted, weight-adjusted, and BMI-adjusted muscle mass indices yield different prevalence estimates and clinical classifications for sarcopenia. Tinggaard et al. [30] found that ALM/BMI was clinically superior to ALM/height^2^ for identifying muscle dysfunction in heart failure patients. Similarly, Bufano et al. [31] reported that sarcopenic obesity prevalence ranged from 2.3% to 47.6% depending on the cut-off method used. In the present study, a methodologically important finding emerged from comparing Figure 6C and Figure 9. When pre-operative fat was measured as a percentage (Figure 6C), no association with ALM loss was detected in females (*p* > 0.05). When measured in kilograms and adjusted for height, age, and sex (Figure 9), the association became significant (*p* < 0.001). The male association was strong in both analyses. Our findings reinforce that the choice of exposure metric, percentage vs. absolute mass, directly affects whether a sex-specific relationship is detected, with implications for study design and clinical screening.

### 4.10. Limitations and Future Directions

Several limitations of this study should be acknowledged. First, the retrospective, single-center design limits generalizability. Second, body composition was assessed by BIA rather than a reference imaging method. Third, only a single post-operative time point was available; whether the lean mass advantage observed in higher-adiposity patients is maintained or eroded beyond one year remains unknown. Fourth, data on protein and total energy intake, adherence to dietary recommendations, physical activity, nutritional supplementation and hormone levels were not available, and these factors may have contributed to the observed changes in ALM as well as confounding the fat-ALM associations observed. Fifth, tests of interaction carry lower statistical power than tests of the corresponding main effects. The sex × fat interaction results should therefore be regarded as exploratory and require confirmation in larger cohorts. Sixth, the cohort was unbalanced by sex (84 women, 37 men). Estimates in the male subgroup are therefore less precise than those in the female subgroup, and small sex-specific effects may have gone undetected in men. Seventh, because no correction for multiple testing was applied, findings with *p* values near 0.05 warrant more cautious interpretation. Eighth, 71 patients who met the inclusion criteria had no one-year post-operative BIA measurement and were therefore not included in the main analysis. Across the whole cohort their baseline characteristics were similar to those of the patients with one-year data in sex distribution, weight, BMI, FM, FFM and ALM, and differed only in age (Table S2). Among men, however, those without a one-year measurement had lower pre-operative weight, BMI and FM, and this is the subgroup in which the fat-ALM association was strongest. Therefore, selection bias cannot be excluded. Ninth, the analyses relating pre-operative fat to ALM (Figure 6, Figure 7, Figure 8 and Figure 9) were exploratory rather than confirmatory. The models are associational and do not establish causality, and the residualization removes the measured influence of height, age and sex but not that of unmeasured factors. These estimates should therefore be read as generating hypotheses for prospective testing rather than as quantifying an effect that can be generalized beyond this cohort.

The mechanism underlying the positive association between pre-operative FM and post-operative ALM remains unknown. Future multicenter studies are needed to elucidate potential underlying mechanisms, including the effects of chronic mechanical loading, fat-soluble vitamin mobilization, hyperinsulinemia, leptin signaling, and energy substrate availability, on ALM before and after surgery in patients with greater pre-operative adiposity.

## 5. Conclusions

Body composition changes following laparoscopic sleeve gastrectomy in this cohort of 121 patients were metric-dependent and more complex than total weight loss alone would suggest. Although absolute ALM declined significantly in both sexes, ALM/weight and ALM/BMI indices rose post-operatively; this was a mathematical consequence of FM declining faster than lean tissue. Males and females differed in both the magnitude and the regional distribution of lean mass loss: males lost proportionally more from the limbs, females from the trunk. Patients who carried more excess fat before surgery had more lean mass both before and one year after surgery, yet lost more lean mass over the surgical year, an effect more pronounced in male patients.

Patients with lower pre-operative fat mass may present at one year with lower post-operative ALM than those with higher pre-operative adiposity. This observation generates the hypothesis for future studies that such patients may benefit from closer post-operative nutritional and rehabilitation monitoring, which was not tested in this study. These findings require prospective validation in larger, multicenter cohorts using imaging-based reference methods.

## Figures and Tables

**Table 1:** Demographic, anthropometric and body composition characteristics by sex, before and one year after LSG.

Characteristic	Overall (*N* = 121)	Female (*n* = 84)	Male (*n* = 37)	*p*-Value
Age (years)	40.1 ± 12.0	40.7 ± 12.3	38.7 ± 11.3	>0.05
Height (m)	1.67 ± 0.09	1.62 ± 0.06	1.77 ± 0.06	<0.001
Pre-operative weight (kg)	119.4 (104.1–138.3)	109.1 (99.9–123.9)	147.6 (134.1–170.8)	<0.001
Post-operative weight (kg)	77.2 (64.9–90.5)	69.2 (60.9–82.6)	94.9 (81.8–104.5)	<0.001
Total weight loss (%)	37.4 (31.3–43.2)	36.4 (31.2–42.6)	38.7 (31.8–44.4)	>0.05
Pre-operative BMI (kg/m^2^)	43.4 (38.5–48.0)	41.6 (38.0–46.3)	47.0 (42.7–52.9)	<0.001
Post-operative BMI (kg/m^2^)	27.1 (24.3–32.0)	25.9 (23.6–30.8)	29.4 (26.5–32.6)	<0.01
Pre-operative FM (kg)	53.1 (44.9–63.2)	49.6 (42.8–57.9)	63.7 (51.5–82.0)	<0.001
Post-operative FM (kg)	20.4 (15.4–27.9)	20.1 (15.6–27.6)	24.7 (15.4–28.0)	>0.05
Pre-operative FFM (kg)	64.1 (57.3–78.4)	60.0 (54.8–65.6)	85.3 (78.9–92.4)	<0.001
Post-operative FFM (kg)	53.3 (46.7–62.6)	49.6 (45.5–54.1)	70.4 (65.8–76.2)	<0.001
Pre-operative ALM (kg)	26.4 (23.2–36.1)	24.1 (22.5–26.7)	42.2 (36.8–46.6)	<0.001
Post-operative ALM (kg)	21.9 (19.2–27.7)	20.2 (18.6–22.0)	31.6 (28.6–35.2)	<0.001

ALM, appendicular lean soft tissue mass; BMI, body mass index; FM, fat mass; FFM, fat-free mass.

**Table 2:** Regional body composition and derived indices by sex, before and one year after LSG.

Variable	Female (*n* = 84) Pre-Operative	Female (*n* = 84) Post-Operative	Male (*n* = 37) Pre-Operative	Male (*n* = 37) Post-Operative
Lean soft tissue mass (kg)				
Trunk	32.85 (29.77–35.02)	26.65 (24.40–29.30)	38.80 (36.40–41.00)	35.10 (32.30–36.40)
Left arm	2.90 (2.70–3.20)	2.25 (2.00–2.60)	5.40 (4.50–5.90)	3.90 (3.60–4.30)
Right arm	2.80 (2.50–2.92)	2.20 (2.00–2.50)	5.40 (4.60–5.90)	3.90 (3.60–4.20)
Left leg	9.25 (8.60–10.12)	7.80 (7.10–8.40)	15.70 (14.50–18.00)	11.60 (10.60–13.20)
Right leg	9.30 (8.60–10.33)	7.90 (7.28–8.53)	15.70 (14.30–17.30)	11.60 (10.90–13.60)
FM (kg)				
Trunk	21.10 (19.00–24.55)	9.05 (6.57–11.80)	34.20 (29.70–44.90)	16.20 (9.90–19.60)
Left arm	4.10 (3.10–5.20)	1.20 (0.90–1.90)	4.50 (3.10–6.40)	1.30 (0.90–1.50)
Right arm	3.65 (2.80–4.60)	1.10 (0.80–1.82)	4.00 (3.00–5.80)	1.20 (0.80–1.40)
Left leg	10.10 (8.38–12.03)	4.05 (3.58–6.08)	9.50 (7.20–11.70)	2.50 (1.90–3.30)
Right leg	10.05 (8.47–12.12)	4.15 (3.60–6.25)	9.80 (7.60–12.40)	2.60 (2.00–3.50)
ALM indices				
ALM/W × 100 (%)	22.24 (21.41–22.96)	29.08 (26.80–31.00)	28.08 (27.25–29.20)	34.42 (32.38–36.08)
ALM/BMI	0.587 (0.549–0.615)	0.767 (0.694–0.824)	0.881 (0.845–0.943)	1.085 (0.993–1.148)
ALM/height^2^ (kg/m^2^)	9.23 (8.61–9.97)	7.64 (7.12–8.28)	13.32 (12.20–15.02)	10.13 (9.50–10.69)
Adiposity indices				
Body fat percentage	45.50 (42.90–47.23)	28.85 (25.18–33.67)	42.80 (38.90–47.30)	24.90 (19.50–28.00)
FM/FFM ratio	0.834 (0.752–0.894)	0.406 (0.337–0.507)	0.749 (0.636–0.899)	0.332 (0.242–0.389)

All values are median (interquartile range); units are given with each variable. Every pre- to post-operative change was significant in both sexes (all *p* < 0.001). Between-sex comparisons are reported in Section 3.3, Section 3.4, Section 3.5 and Section 3.6. ALM, appendicular lean soft tissue mass; BMI, body mass index.

**Table 3:** Sex-stratified linear regression of appendicular lean soft tissue mass on pre-operative adiposity (Figure 6, Figure 7, Figure 8 and Figure 9).

Group	b (95% CI)	r	*p*
Figure 6A. Pre-operative ALM vs. body fat %, unadjusted			
Female	+0.249 (+0.035 to +0.462)	+0.248	<0.05
Male	+0.916 (+0.553 to +1.279)	+0.655	<0.001
Figure 6B. Post-operative ALM vs. pre-operative body fat %, unadjusted			
Female	+0.259 (+0.113 to +0.405)	+0.364	<0.001
Male	+0.370 (+0.125 to +0.615)	+0.460	<0.01
Figure 6C. Change in ALM vs. pre-operative body fat %, unadjusted			
Female	+0.010 (−0.104 to +0.125)	+0.020	>0.05
Male	−0.546 (−0.734 to −0.357)	−0.704	<0.001
Figure 7. Pre-operative ALM vs. pre-operative FM, residualized			
Overall	+0.288 (+0.250 to +0.327)	+0.808	<0.001
Female	+0.231 (+0.183 to +0.280)	+0.725	<0.001
Male	+0.320 (+0.252 to +0.388)	+0.851	<0.001
Figure 8. Post-operative ALM vs. pre-operative FM, residualized			
Overall	+0.154 (+0.125 to +0.182)	+0.704	<0.001
Female	+0.159 (+0.125 to +0.193)	+0.715	<0.001
Male	+0.150 (+0.097 to +0.203)	+0.697	<0.001
Figure 9. Change in ALM vs. pre-operative FM, residualized			
Overall	−0.135 (−0.161 to −0.108)	−0.680	<0.001
Female	−0.072 (−0.107 to −0.038)	−0.419	<0.001
Male	−0.170 (−0.213 to −0.126)	−0.803	<0.001

b is expressed in kilograms of ALM per one percentage point of pre-operative body fat for Figure 6, and in kilograms of ALM per kilogram of pre-operative FM for Figure 7, Figure 8 and Figure 9. ALM, appendicular lean soft tissue mass; CI, confidence interval.

## Data Availability

The data presented in this study are available on request from the corresponding author. The data are not publicly available due to privacy and ethical restrictions.

## Supplementary Materials

The following supporting information can be downloaded at: https://www.mdpi.com/article/10.3390/diagnostics16162656/s1, Figure S1: Patient flow diagram. Table S1: Statistical test applied to each comparison reported in Table 1 and Figure 1, Figure 2, Figure 3, Figure 4 and Figure 5. Table S2: Baseline characteristics of the patients who met the inclusion criteria and underwent LSG, according to whether a one-year post-operative BIA measurement was available, overall and by sex.

## Abbreviations

The following abbreviations are used in this manuscript:

ALM	Appendicular lean soft tissue mass
BIA	Bioelectrical impedance analysis
BMI	Body mass index
CI	Confidence interval
CT	Computed tomography
DXA	Dual-energy X-ray absorptiometry
EASO	European Association for the Study of Obesity
ESPEN	European Society for Clinical Nutrition and Metabolism
FFM	Fat-free mass
FM	Fat mass
HC3	Heteroscedasticity-consistent (type 3) standard errors
IQR	Interquartile range
LSG	Laparoscopic sleeve gastrectomy
LST	Lean soft tissue
MRI	Magnetic resonance imaging
OLS	Ordinary least squares
SD	Standard deviation
SMM	Skeletal muscle mass
