# Supplementary material for: Association Between Pre-Operative Fat Mass and Appendicular Lean Soft-Tissue Mass Before and One Year After Laparoscopic Sleeve Gastrectomy: A Sex-Stratified Body Composition Study
Source: Diagnostics (Basel). 2026 Aug 20;16(16):2656. doi: 10.3390/diagnostics16162656 (PMC13512490; doi:10.3390/diagnostics16162656)
Supplement: Supplementary file 1 [file diagnostics-16-02656-s001.zip › Table S1.pdf]

Table S1. Statistical test applied to each comparison reported in Table 1 and Figures 1 to 5.

Figure or table	Variable	Comparison	Test	p-value
Table 1				
Table 1	Age	Female vs male	Welch's t-test	$p > 0.05$
Table 1	Height	Female vs male	Welch's t-test	$p < 0.001$
Table 1	Pre-operative weight	Female vs male	Mann-Whitney U test	$p < 0.001$
Table 1	Post-operative weight	Female vs male	Mann-Whitney U test	$p < 0.001$
Table 1	Pre-operative BMI	Female vs male	Mann-Whitney U test	$p < 0.001$
Table 1	Post-operative BMI	Female vs male	Mann-Whitney U test	$p < 0.01$
Table 1	Pre-operative fat mass	Female vs male	Mann-Whitney U test	$p < 0.001$
Table 1	Pre-operative ALM	Female vs male	Mann-Whitney U test	$p < 0.001$
Figure 1				
Figure 1A	Body mass index	Female, pre- vs post-operative	Wilcoxon signed-rank test	$p < 0.001$
Figure 1A	Body mass index	Male, pre- vs post-operative	Paired t-test	$p < 0.001$
Figure 1A	Body mass index	Female vs male, pre-operative	Mann-Whitney U test	$p < 0.001$
Figure 1A	Body mass index	Female vs male, post-operative	Mann-Whitney U test	$p < 0.01$
Figure 1A	Body mass index	Female vs male, absolute change	Mann-Whitney U test	$p < 0.01$
Figure 1B	Total weight loss percentage	Female vs male	Mann-Whitney U test	$p > 0.05$
Figure 1C	Fat-free mass	Female, pre- vs post-operative	Paired t-test	$p < 0.001$
Figure 1C	Fat-free mass	Male, pre- vs post-operative	Paired t-test	$p < 0.001$
Figure 1C	Fat-free mass	Female vs male, pre-operative	Mann-Whitney U test	$p < 0.001$
Figure 1C	Fat-free mass	Female vs male, post-operative	Mann-Whitney U test	$p < 0.001$
Figure 1C	Fat-free mass	Female vs male, absolute change	Welch's t-test	$p < 0.001$
Figure 1C	Fat-free mass	Female vs male, proportional change	Welch's t-test	$p > 0.05$
Figure 1D	Fat mass	Female, pre- vs post-operative	Paired t-test	$p < 0.001$
Figure 1D	Fat mass	Male, pre- vs post-operative	Paired t-test	$p < 0.001$
Figure 1D	Fat mass	Female vs male, pre-operative	Mann-Whitney U test	$p < 0.001$
Figure 1D	Fat mass	Female vs male, post-operative	Mann-Whitney U test	$p > 0.05$
Figure 1D	Fat mass	Female vs male, absolute change	Welch's t-test	$p < 0.001$
Figure 2				
Figure 2A	Trunk lean soft tissue mass	Female, pre- vs post-operative	Paired t-test	$p < 0.001$
Figure 2A	Trunk lean soft tissue mass	Male, pre- vs post-operative	Wilcoxon signed-rank test	$p < 0.001$
Figure 2A	Trunk lean soft tissue mass	Female vs male, pre-operative	Mann-Whitney U test	$p < 0.001$
Figure 2A	Trunk lean soft tissue mass	Female vs male, post-operative	Mann-Whitney U test	$p < 0.001$
Figure 2A	Trunk lean soft tissue mass	Female vs male, proportional change	Mann-Whitney U test	$p < 0.001$
Figure 2B	Left arm lean soft tissue mass	Female, pre- vs post-operative	Paired t-test	$p < 0.001$
Figure 2B	Left arm lean soft tissue mass	Male, pre- vs post-operative	Paired t-test	$p < 0.001$
Figure 2B	Left arm lean soft tissue mass	Female vs male, pre-operative	Mann-Whitney U test	$p < 0.001$
Figure 2B	Left arm lean soft tissue mass	Female vs male, post-operative	Mann-Whitney U test	$p < 0.001$
Figure 2B	Left arm lean soft tissue mass	Female vs male, proportional change	Mann-Whitney U test	$p < 0.05$
Figure 2B	Right arm lean soft tissue mass	Female, pre- vs post-operative	Wilcoxon signed-rank test	$p < 0.001$
Figure 2B	Right arm lean soft tissue mass	Male, pre- vs post-operative	Paired t-test	$p < 0.001$
Figure 2B	Right arm lean soft tissue mass	Female vs male, pre-operative	Mann-Whitney U test	$p < 0.001$
Figure 2B	Right arm lean soft tissue mass	Female vs male, post-operative	Mann-Whitney U test	$p < 0.001$
Figure 2B	Right arm lean soft tissue mass	Female vs male, proportional change	Welch's t-test	$p < 0.001$
Figure 2A	Left leg lean soft tissue mass	Female, pre- vs post-operative	Wilcoxon signed-rank test	$p < 0.001$
Figure 2A	Left leg lean soft tissue mass	Male, pre- vs post-operative	Paired t-test	$p < 0.001$

Figure 2A	Left leg lean soft tissue mass	Female vs male, pre-operative	Mann-Whitney U test	$p < 0.001$
Figure 2A	Left leg lean soft tissue mass	Female vs male, post-operative	Mann-Whitney U test	$p < 0.001$
Figure 2A	Left leg lean soft tissue mass	Female vs male, proportional change	Welch's t-test	$p < 0.001$
Figure 2A	Right leg lean soft tissue mass	Female, pre- vs post-operative	Wilcoxon signed-rank test	$p < 0.001$
Figure 2A	Right leg lean soft tissue mass	Male, pre- vs post-operative	Paired t-test	$p < 0.001$
Figure 2A	Right leg lean soft tissue mass	Female vs male, pre-operative	Mann-Whitney U test	$p < 0.001$
Figure 2A	Right leg lean soft tissue mass	Female vs male, post-operative	Mann-Whitney U test	$p < 0.001$
Figure 2A	Right leg lean soft tissue mass	Female vs male, proportional change	Welch's t-test	$p < 0.001$
Figure 2A	Trunk lean soft tissue mass	Female vs male, absolute change	Mann-Whitney U test	$p < 0.001$
Figure 3				
Figure 3A	Trunk fat mass	Female, pre- vs post-operative	Paired t-test	$p < 0.001$
Figure 3A	Trunk fat mass	Male, pre- vs post-operative	Paired t-test	$p < 0.001$
Figure 3A	Trunk fat mass	Female vs male, pre-operative	Mann-Whitney U test	$p < 0.001$
Figure 3A	Trunk fat mass	Female vs male, post-operative	Mann-Whitney U test	$p < 0.001$
Figure 3A	Trunk fat mass	Female vs male, proportional change	Mann-Whitney U test	$p > 0.05$
Figure 3B	Left arm fat mass	Female, pre- vs post-operative	Wilcoxon signed-rank test	$p < 0.001$
Figure 3B	Left arm fat mass	Male, pre- vs post-operative	Wilcoxon signed-rank test	$p < 0.001$
Figure 3B	Left arm fat mass	Female vs male, pre-operative	Mann-Whitney U test	$p > 0.05$
Figure 3B	Left arm fat mass	Female vs male, post-operative	Mann-Whitney U test	$p > 0.05$
Figure 3B	Left arm fat mass	Female vs male, proportional change	Mann-Whitney U test	$p > 0.05$
Figure 3B	Right arm fat mass	Female, pre- vs post-operative	Wilcoxon signed-rank test	$p < 0.001$
Figure 3B	Right arm fat mass	Male, pre- vs post-operative	Paired t-test	$p < 0.001$
Figure 3B	Right arm fat mass	Female vs male, pre-operative	Mann-Whitney U test	$p > 0.05$
Figure 3B	Right arm fat mass	Female vs male, post-operative	Mann-Whitney U test	$p > 0.05$
Figure 3B	Right arm fat mass	Female vs male, proportional change	Mann-Whitney U test	$p > 0.05$
Figure 3A	Left leg fat mass	Female, pre- vs post-operative	Wilcoxon signed-rank test	$p < 0.001$
Figure 3A	Left leg fat mass	Male, pre- vs post-operative	Paired t-test	$p < 0.001$
Figure 3A	Left leg fat mass	Female vs male, pre-operative	Mann-Whitney U test	$p > 0.05$
Figure 3A	Left leg fat mass	Female vs male, post-operative	Mann-Whitney U test	$p < 0.001$
Figure 3A	Left leg fat mass	Female vs male, proportional change	Welch's t-test	$p < 0.001$
Figure 3A	Right leg fat mass	Female, pre- vs post-operative	Wilcoxon signed-rank test	$p < 0.001$
Figure 3A	Right leg fat mass	Male, pre- vs post-operative	Paired t-test	$p < 0.001$
Figure 3A	Right leg fat mass	Female vs male, pre-operative	Mann-Whitney U test	$p > 0.05$
Figure 3A	Right leg fat mass	Female vs male, post-operative	Mann-Whitney U test	$p < 0.001$
Figure 3A	Right leg fat mass	Female vs male, proportional change	Mann-Whitney U test	$p < 0.001$
Figure 3A	Trunk fat mass	Female vs male, absolute change	Welch's t-test	$p < 0.001$
Figure 4				
Figure 4A	ALM/W x 100	Female, pre- vs post-operative	Paired t-test	$p < 0.001$
Figure 4A	ALM/W x 100	Male, pre- vs post-operative	Paired t-test	$p < 0.001$
Figure 4A	ALM/W x 100	Female vs male, pre-operative	Mann-Whitney U test	$p < 0.001$
Figure 4A	ALM/W x 100	Female vs male, post-operative	Welch's t-test	$p < 0.001$
Figure 4B	ALM/BMI	Female, pre- vs post-operative	Paired t-test	$p < 0.001$
Figure 4B	ALM/BMI	Male, pre- vs post-operative	Paired t-test	$p < 0.001$
Figure 4B	ALM/BMI	Female vs male, pre-operative	Mann-Whitney U test	$p < 0.001$

Figure 4B	ALM/BMI	Female vs male, post-operative	Welch's t-test	$p < 0.001$
Figure 4C	ALM/height^2	Female, pre- vs post-operative	Wilcoxon signed-rank test	$p < 0.001$
Figure 4C	ALM/height^2	Male, pre- vs post-operative	Paired t-test	$p < 0.001$
Figure 4C	ALM/height^2	Female vs male, pre-operative	Mann-Whitney U test	$p < 0.001$
Figure 4C	ALM/height^2	Female vs male, post-operative	Mann-Whitney U test	$p < 0.001$
Figure 5				
Figure 5A	Body fat percentage	Female, pre- vs post-operative	Paired t-test	$p < 0.001$
Figure 5A	Body fat percentage	Male, pre- vs post-operative	Paired t-test	$p < 0.001$
Figure 5A	Body fat percentage	Female vs male, pre-operative	Welch's t-test	$p < 0.05$
Figure 5A	Body fat percentage	Female vs male, post-operative	Welch's t-test	$p < 0.001$
Figure 5A	Body fat percentage	Female vs male, relative reduction	Welch's t-test	$p < 0.01$
Figure 5B	Fat mass / fat-free mass ratio	Female, pre- vs post-operative	Paired t-test	$p < 0.001$
Figure 5B	Fat mass / fat-free mass ratio	Male, pre- vs post-operative	Paired t-test	$p < 0.001$
Figure 5B	Fat mass / fat-free mass ratio	Female vs male, pre-operative	Mann-Whitney U test	$p < 0.05$
Figure 5B	Fat mass / fat-free mass ratio	Female vs male, post-operative	Mann-Whitney U test	$p < 0.001$
Figure 5B	Fat mass / fat-free mass ratio	Female vs male, relative reduction	Mann-Whitney U test	$p < 0.01$

Tests follow the convention stated in Section 2.4. Within-sex pre- to post-operative comparisons used the paired t-test when the difference scores were normally distributed and the Wilcoxon signed-rank test otherwise. Between-sex comparisons used Welch's independent t-test when both groups were normally distributed and the Mann-Whitney U test otherwise. Normality was assessed with the Shapiro-Wilk test in each sex separately. All comparisons made in the manuscript are listed, whether or not they reached statistical significance. Estimates and p-values for the sex-stratified regression models in Figures 6 to 9 are reported in Table 3. ALM, appendicular lean soft tissue mass; BMI, body mass index; LST, lean soft tissue.
