# Supplementary material for: Association Between Pre-Operative Fat Mass and Appendicular Lean Soft-Tissue Mass Before and One Year After Laparoscopic Sleeve Gastrectomy: A Sex-Stratified Body Composition Study
Source: Diagnostics (Basel). 2026 Aug 20;16(16):2656. doi: 10.3390/diagnostics16162656 (PMC13512490; doi:10.3390/diagnostics16162656)
Supplement: Supplementary file 1 [file diagnostics-16-02656-s001.zip › Table S2.pdf]

Table S2. Baseline characteristics of the patients who met the inclusion criteria and underwent LSG, according to whether a one-year post-operative BIA measurement was available, overall and by sex.

**Characteristic**	**With one-year BIA data**<br>***N = 121***	**Without one-year BIA data**<br>***N = 71***	**Test**	**p-value**
Comparisons of all eligible patients				
Female, n (%)	84 (69.4%)	41 (57.7%)	Chi-squared	> 0.05
Age (years)	41.0 (30.0–49.0)	34.0 (26.5–42.0)	Mann-Whitney U	< 0.01
Height (m)	1.67 ± 0.09	1.70 ± 0.09	Welch's t-test	< 0.05
Pre-operative weight (kg)	119.4 (104.1–138.3)	126.7 (106.3–140.4)	Mann-Whitney U	> 0.05
Pre-operative BMI (kg/m²)	43.4 (38.5–48.0)	41.8 (38.8–46.6)	Mann-Whitney U	> 0.05
Pre-operative fat mass (kg)	53.1 (44.9–63.2)	51.7 (44.3–60.1)	Mann-Whitney U	> 0.05
Pre-operative fat-free mass (kg)	64.1 (57.3–78.4)	68.9 (59.0–83.0)	Mann-Whitney U	> 0.05
Pre-operative ALM (kg)	26.4 (23.2–36.1)	28.0 (23.8–40.1)	Mann-Whitney U	> 0.05
Comparisons of Female Patients	**With one-year BIA data**<br>***n = 84***	**Without one-year BIA data**<br>***n = 41***	**Test**	**p-value**
Age (years)	41.0 (30.0–51.2)	34.0 (26.0–41.0)	Mann-Whitney U	< 0.05
Height (m)	1.62 ± 0.06	1.64 ± 0.06	Welch's t-test	> 0.05
Pre-operative weight (kg)	109.1 (99.9–123.9)	108.9 (99.5–119.4)	Mann-Whitney U	> 0.05
Pre-operative BMI (kg/m²)	41.6 (38.0–46.3)	39.4 (37.5–43.9)	Mann-Whitney U	> 0.05
Pre-operative fat mass (kg)	49.6 (42.8–57.9)	50.3 (42.6–55.2)	Mann-Whitney U	> 0.05
Pre-operative fat-free mass (kg)	60.0 (54.8–65.6)	59.6 (56.1–62.9)	Mann-Whitney U	> 0.05
Pre-operative ALM (kg)	24.1 (22.5–26.7)	24.2 (23.0–26.0)	Mann-Whitney U	> 0.05
Comparisons of Male Patients	**With one-year BIA data**<br>**n = 37**	**Without one-year BIA data**<br>**n = 30**	**Test**	**p-value**
Age (years)	38.7 ± 11.3	35.4 ± 10.1	Welch's t-test	> 0.05
Height (m)	1.77 ± 0.06	1.79 ± 0.06	Welch's t-test	> 0.05
Pre-operative weight (kg)	152.7 ± 28.1	141.6 ± 13.5	Welch's t-test	< 0.05
Pre-operative BMI (kg/m²)	48.7 ± 8.7	44.5 ± 3.8	Welch's t-test	< 0.01
Pre-operative fat mass (kg)	67.3 ± 20.7	56.8 ± 9.0	Welch's t-test	< 0.01
Pre-operative fat-free mass (kg)	85.3 (78.9–92.4)	84.2 (81.5–86.8)	Mann-Whitney U	> 0.05
Pre-operative ALM (kg)	42.2 (36.8–46.6)	40.9 (39.3–42.1)	Mann-Whitney U	> 0.05

Both groups are drawn from the 192 patients who met the inclusion criteria and underwent LSG. Those with a one-year BIA measurement (n = 121) were included in the main analysis; those without one (n = 71) have no measurement at the one-year timepoint within the study period. Patients excluded by design are not included here. Tests follow the convention stated in Section 2.4: normality was assessed with the Shapiro-Wilk test in each group, and comparisons used Welch's independent t-test when both groups were normally distributed and the Mann-Whitney U test otherwise. Values are median (interquartile range) except where both groups were normally distributed, in which case mean ± standard deviation is given. Sex was compared with the chi-squared test. ALM was calculated as the sum of the lean soft tissue mass of both arms and both legs. The height difference in the all-patients panel reflects the sex composition of the two groups and is not significant within either sex. ALM, appendicular lean soft tissue mass; BMI, body mass index; LSG, laparoscopic sleeve gastrectomy.
